# ﻿*Biconidiumsinense* gen. et sp. nov. (Hypocreales, Bionectriaceae) and *Didymocyrtisshanxiensis* sp. nov. (Phaeosphaeriaceae, *Didymocyrtis*) isolated from urban soil in China

**DOI:** 10.3897/mycokeys.116.146683

**Published:** 2025-04-29

**Authors:** Hai-Yan Wang, Chunbo Dong, Yan-Wei Zhang, Wan-Hao Chen, Yan-Feng Han

**Affiliations:** 1 Institute of Fungus Resources, Department of Ecology, College of Life Science, Guizhou University, Guiyang 550025, Guizhou, China Guizhou University Guiyang China; 2 Key Laboratory of Development and Utilization of Biological Resources in Colleges and Universities of Guizhou Province/Key Laboratory of Ecology and Management on Forest Fire in Higher Education Institutions of Guizhou Province, Guizhou Education University, Guiyang 550018, Guizhou, China Guizhou Education University Guiyang China; 3 Center for Mycomedicine Research, Basic Medical School, Guizhou University of Traditional Chinese Medicine, Guiyang 550025, Guizhou, China Guizhou University of Traditional Chinese Medicine Guiyang China

**Keywords:** Fungal taxonomy, mycodiversity, new taxa, phylogeny

## Abstract

During a fungal diversity survey in various urban habitats across China, 5 fungal isolates were discovered from soil samples. Detailed morphological observations and multi-gene phylogenetic analyses confirmed the identification of two novel taxa: *Biconidiumsinense***gen. et sp. nov.** and *Didymocyrtisshanxiensis***sp. nov.** These species were formally described, illustrated, and discussed, highlighting their distinct characteristics and taxonomic placement. The study expands our understanding of fungal diversity in urban environments, emphasizing the importance of combining morphological and molecular approaches for accurate species delineation and discovery.

## ﻿Introduction

Soil fungi play an important role in mediating the processes of geochemical cycling, ecosystem material cycling and energy flow. For instance, they influence soil fertility, mineral breakdown, and organic matter cycling, as well as plant health and nutrition (Guo et al. 2017; Lu 2018). Moreover, some soil fungi can produce a lot of metabolites that are essential for human life and production (Zhang et al. 2023; Wang et al. 2024). For example, among the fungal species in the soil, the strains of the genera *Aspergillus*, *Penicillium*, *Paecilomyces* and *Trichoderma* produce flavins, ankaflavin, quinones, and anthraquinone (Akilandeswari and Pradeep 2016). *Penicilliumgriseofulvum* can produce a range of secondary metabolites including chanoclavine I, elymoclavine, fulvic acid, and griseofulvin, all of which can be used for antimicrobial activity (Yogabaanu et al. 2017). *Acrophialophoralevis* QHDZ1–2 isolated from a zoo soil can produce some compounds, such as amino acid, amines, fatty acid, and vitamins (Wang 2024). Due to a multitude of factors, it is suspected that species are disappearing before they are discovered in many habitats (Wang et al. 2018; Löbl et al. 2023; Wang et al. 2024). This implies that we still need to make more efforts to delve deeper into soil fungi resources, contribute to the study of our Earth’s fungal diversity, and provide fungal resources for social industrial production. Currently, numerous studies have investigated fungal diversity in various soil habitats across China, including caves, forests, farmland, deserts and grasslands (Guo et al. 2017; Ma et al. 2021; Ma 2023; Guo 2024; Song et al. 2024). However, the composition and diversity of soil fungi in various urban various environments appear to have been neglected.

Urbanization has been the most impactful human activity in altering landscape patterns over the past century and is widely regarded as a significant threat to global biodiversity (Grimm et al. 2008; Nugent and Allison 2022). Developing countries are experiencing the swiftest rates of urbanization, with projections indicating that approximately 68% of the global population will reside in urban areas by 2050 (Desa 2019). The process of urbanization will reshape the land landscape, impacting elements such as surface vegetation, hydrology and soil, which in turn affects biodiversity and can lead to species homogenization or even extinction of species (Buczkowski and Richmond 2012; Yan et al. 2022). Urbanization has had a profound impact on soil fungi. It fragments the original habitats, resulting in a decline in fungal diversity and the potential disappearance of some native fungal species (Zhao et al. 2012; Hou et al. 2014; Rai et al. 2018). Consequently, in the context of urbanization, the composition and distribution of soil fungi across various urban habitats should be paid more attention. In recent years, the composition and diversity of green soil fungi in different urban habitats were explored (Zhang et al. 2021, 2023, 2024; Li et al. 2022a, 2022b; Ren et al. 2022; Wang et al. 2023, 2024). Fortunately, many new species and genera have been discovered and documented in these urban settings.

Bionectriaceae Samuels & Rossman was proposed by Rossman et al. (1999) based on the sexual morph-typified genus *Bionectria* Speg. (Spegazzini 1919). It is including 26 genera. Its diagnostic characteristics are the presence of white, pale tan orange or brown, uniloculate, perithecial, rarely cleistothecial ascomata and generally not changing color in KOH.

Barr (1979) proposed the family Phaeosphaeriaceae using *Phaeosphaeria* with *Ph.oryzae* as the type species. The new genus *Diederichomyces* was described by Trakunyingcharoen et al. (2014) to include most of the lichenicolous *Phoma* species that were assigned to the Phaeosphaeriaceae by Lawrey et al. (2012). Vainio (1921) established *Didymocyrtis* Vain., based on the type species *Didymocyrtisconsimilis* Vain. With the development of phylogeny, the lichenicolous species of genus *Didymocyrtis* had been assigned to *Diederichia* D. Hawksw., *Diederichomyces* Crous & Trakun., *Leptosphaeria* Pass. and *Phoma* Sacc. (Trakunyingcharoen et al. 2014). Recently, the genus *Didymocyrtis* was resurrected for these species, and the new combinations, *Didymocyrtisbryonthae* (Arnold) Hafellner, *Didymocyrtiscladoniicola* (Diederich, Kocourk. & Etayo) Ertz & Diederich, *Didymocyrtisfoliaceiphila* (Diederich, Kocourk. & Etayo) Ertz & Diederich, *Didymocyrtisinfestans* (Speg.) Hafellner, *Didymocyrtiskaernefeltii* (S.Y. Kondr.) Hafellner, *Didymocyrtismelanelixiae* (Brackel) Diederich, R.C. Harris & Etayo, *Didymocyrtispseudeverniae* (Etayo & Diederich) Ertz & Diederich, *Didymocyrtisramalinae* (Roberge ex Desm.) Ertz, Diederich & Hafellner, *Didymocyrtisslaptoniensis* (D. Hawksw.) Hafellner & Ertz, and *Didymocyrtisxanthomendozae* (Diederich & Freebury) Diederich & Freebury were created (Ertz et al. 2015). Presently, the genus *Didymocyrtis* includes twenty-nine species in the Index Fungorum.

During a continuous survey of fungal diversity exploration from different urban green soils in China, five strains from green soils of sewage treatment plant were isolated and purified. Based on the multi-gene phylogeny and morphological characteristics, these isolated strains were identified as two new taxa, *Biconidiumsinense* gen. et sp. nov. and *Didymocyrtisshanxiensis* sp. nov., which are described and illustrated.

## ﻿Materials and methods

### ﻿Sample collection and fungal isolation

Soil samples, from 3–10 cm below the soil surface, were collected from green soil of sewage treatment plant in some cities in China. Samples were placed in sterile Ziploc plastic bags, and brought back to the laboratory. Then, the 2 g of each soil samples for fungal isolation, were placed into a sterile conical flask containing 20 mL sterile water in a 50 mL sterile conical flask, and thoroughly shaken using a Vortex vibration meter. Subsequently, the soil suspension was diluted to a concentration of 10^-3^. Then, 1 mL of the diluted sample was transferred to a sterile Petri dish with Sabouraud’s dextrose agar (SDA; peptone 10 g/L, dextrose 40 g/L, agar 20 g/L, 3.3 mL of 1% Bengal red aqueous solution) medium containing 50 mg/L penicillin and 50 mg/L streptomycin. The plates were incubated at 25 °C for 1 week, then every single colony was selected from the plates and transferred to new potato dextrose agar (PDA, potato 200 g/L, dextrose 20 g/L, agar 20 g/L) plates.

### ﻿Morphological study

Strains of potentially new species were transferred to plates of malt extract agar (MEA), oatmeal agar (OA) and potato dextrose agar (PDA), and were incubated at 25 °C for examining their colony morphology and microscopic morphology. After 7 days, the colony colors according to national standard color card and diameters on the surface and reverse of inoculated Petri dishes were observed and recorded. Meanwhile, fungal hyphae and conidiogenous structures were examined, and images were captured by making direct wet mounts with 25% lactic acid on PDA, with an optical microscope (DM4 B, Leica). Strains of two novel species were deposited in the Institute of Fungus Resources, Guizhou University (GZUIFR = GZAC). Taxonomic descriptions and nomenclature of one new genus and two new species were uploaded in MycoBank (https://www.mycobank.org/).

### ﻿DNA extraction, PCR amplification and sequencing

Using the BioTeke Fungus Genomic DNA Extraction kit (DP2032, BioTeke), total genomic DNA was extracted following the manufacturer’s instruction. The extracted DNA was stored at −20 °C. Primer combinations: ITS1/ITS4 (White et al. 1990), LR0R/LR5 (Wang et al. 2022) and T1/TUB4Rd (O’Donnell and Cigelnik 1997; Woudenberg et al. 2009) were used for amplification of the internal transcribed spacers (ITS), the 28S nrRNA locus (LSU) and beta-tubulin gene (*tub2*), respectively. The PCR amplification conditions: ITS, 94 °C: 5 min, (94 °C: 30 s, 51 °C: 50 s, 72 °C: 45 s) × 35 cycles, 72 °C: 10 min (White et al. 1990); LSU, 94 °C: 5 min, (94 °C: 30 s, 51 °C: 1 min, 72 °C: 2 min) × 35 cycles, 72 °C: 10 min (Zhang et al. 2023); *tub2*, 94 °C: 5 min, (94 °C: 30 s, 52 °C: 30 s, 72 °C: 30 s) × 35 cycles 72 °C: 10 min (Woudenberg et al. 2009). In this study, the PCR products were sent to Quintarabio (Wuhan, China) for purification and sequencing. Strains sequences of two new species were submitted to GenBank (https://www.ncbi.nlm.nih.gov/) (Table 1 and Table 2).

**Table 1.** Strains of Bionectriaceae and corresponding GenBank numbers included in phylogenetic analyses.

Species	Strains	ITS	LSU	Reference
*Gliomastixmurorum*	CBS 154.25T	OQ429613	HQ232063	Hou et al. (2023)
*Gliomastixmurorum*	CBS 253.79	OQ429614	OQ055521	Hou et al. (2023)
*Gliomastixroseogrisea*	CBS 134.56T	OQ429639	OQ055545	Hou et al. (2023)
*Gliomastixtumulicola*	CBS 127532T	OQ429641	OQ055547	Hou et al. (2023)
*Paracylindrocarponaloicola*	CBS 141300T	KX228277	KX228328	Hou et al. (2023)
*Paracylindrocarponaloicola*	CBS 135907	OQ429762	OQ055661	Hou et al. (2023)
*Paracylindrocarponaurantiacum*	CBS 135909T	OQ429763	OQ055662	Hou et al. (2023)
*Paracylindrocarponmultiseptatum*	CBS 337.77T	OQ429768	OQ055666	Hou et al. (2023)
*Fusariellacurvata*	MFLUCC 15-0844T	KX025152	KX025154	Hou et al. (2023)
*Fusariellaatrovirens*	CBS 311.73	OQ429594	OR052105	Hou et al. (2023)
*Fusariellaarenula*	CBS 330.77	OQ429592	OQ055503	Hou et al. (2023)
*Fusariellaarenula*	CBS 329.77	OQ429593	OQ055504	Hou et al. (2023)
*Seliniapulchra*	A.R. 2812	HM484859	GQ505992	Hou et al. (2023)
*Roumegueriellarufula*	CBS 346.85	OQ429827	OQ430088	Hou et al. (2023)
*Verrucostomamartinicense*	CBS 138731T	OQ429934	OR052121	Hou et al. (2023)
*Verrucostomafreycinetiae*	MAFF 240100T	HM484866	GQ506013	Hou et al. (2023)
*Synnemellisiaaurantia*	COAD 2070 T	KX866395	KX866396	Hou et al. (2023)
*Musananaesporiumtectonae*	CBS 725.87T	OQ429714	OQ055615	Hou et al. (2023)
*Gossypinidiumsporodochiale*	CBS 101694T	OQ429643	OQ055549	Hou et al. (2023)
*Caespitomoniumsquamicola*	CBS 701.73	OQ429515	OQ055426	Hou et al. (2023)
*Caespitomoniumsquamicola*	CBS 392.73	OQ429514	OQ055425	Hou et al. (2023)
*Monohydropisphaerafusigera*	CBS 124147T	OQ429713	OQ055614	Hou et al. (2023)
*Hydropisphaerafungicola*	CBS 122304T	OQ429666	OR052107	Hou et al. (2023)
*Hydropisphaerasuffulta*	CBS 122.87	OQ429672	OQ055577	Hou et al. (2023)
*Paragliomastixrosea*	CBS 277.80AT	OQ429775	OQ055673	Hou et al. (2023)
*Paragliomastixchiangraiensis*	MFLUCC 14-0397T	MN648324	MN648329	Hou et al. (2023)
*Septofusidiumberolinense*	CBS 731.70	OQ429859	OQ430110	Hou et al. (2023)
*Pseudoacremoniumsacchari*	CBS 137990T	KJ869144	KJ869201	Hou et al. (2023)
*Lasionectriaolida*	CBS 799.69T	OQ429693	OQ055598	Hou et al. (2023)
*Lasionectriaolida*	CBS 798.69	OQ429692	OQ055597	Hou et al. (2023)
*Lasionectriacastaneicola*	CBS 122792T	OQ429680	OQ055585	Hou et al. (2023)
*Lasionectriaatrorubra*	CBS 123502T	OQ429674	OQ055579	Hou et al. (2023)
*Verruciconidiapersicina*	CBS 310.59T	OQ429921	OQ430172	Hou et al. (2023)
*Verruciconidiapersicina*	CBS 113716	OQ429922	OQ430173	Hou et al. (2023)
*Verruciconidiaerythroxyli*	CBS 728.87T	OQ429910	OQ430161	Hou et al. (2023)
*Verruciconidiainfuscata*	CBS 100888T	OQ429911	OQ430162	Hou et al. (2023)
*Verruciconidiaquercina*	CBS 469.67T	OQ429925	OQ430176	Hou et al. (2023)
*Verruciconidiaquercina*	CBS 355.77	OQ429927	OQ430178	Hou et al. (2023)
*Lasionectriopsisdentifera*	CBS 650.75	OQ429700	OQ055602	Hou et al. (2023)
*Lasionectriopsisdentifera*	CBS 574.76T	KY607540	KY607555	Hou et al. (2023)
*Ochronectriathailandica*	MFLUCC 15-0140T	KU564071	KU564069	Hou et al. (2023)
*Lasionectriopsisgermanica*	CBS 143538T	OQ429701	MK276528	Hou et al. (2023)
*Ochronectriacalami*	CBS 134535	OQ429755	OQ055654	Hou et al. (2023)
*Lasionectriellaarenuloides*	CBS 576.76T	OQ429696	OQ055601	Hou et al. (2023)
*Lasionectriellamarigotensis*	CBS 131606T	OQ429698	KR105613	Hou et al. (2023)
*Lasionectriellarubioi*	CBS 140157T	OQ429699	KU593581	Hou et al. (2023)
*Ramosiphorumpolyporicola*	CBS 123779T	OQ429823	OQ430084	Hou et al. (2023)
*Ramosiphorumpolyporicola*	CBS 109.87	OQ429822	OQ430083	Hou et al. (2023)
*Ramosiphorumthailandicum*	CBS 101914T	OQ429825	OQ430086	Hou et al. (2023)
*Protocreopsisrutila*	CBS 396.66T	OQ429814	OQ430077	Hou et al. (2023)
*Protocreopsisrutila*	CBS 229.70	OQ429813	OQ430076	Hou et al. (2023)
*Protocreopsisfinnmarkica*	CBS 147428T	OQ429803	OQ055699	Hou et al. (2023)
*Protocreopsisphormiicola*	CBS 567.76T	OQ429806	OQ430069	Hou et al. (2023)
*Protocreopsisfreycinetiae*	CBS 573.76T	OQ429804	OR052113	Hou et al. (2023)
*Nectriopsislindauiana*	CBS 897.70T	OQ429729	OQ055629	Hou et al. (2023)
*Nectriopsisfuliginicola*	CBS 400.82T	KU382175	OQ055628	Hou et al. (2023)
*Nectriopsisviolacea*	CBS 914.70T	OQ429733	OQ055632	Hou et al. (2023)
*Nectriopsisviolacea*	CBS 849.70	OR050510	MH871773	Hou et al. (2023)
*Nectriopsissporangiicola*	CBS 166.74T	AF210661	AF210662	Hou et al. (2023)
*Clonostachysspinulosispora*	CBS 133762T	MH634702	KY006568	Hou et al. (2023)
*Clonostachysphyllophila*	CBS 921.97T	AF210664	OQ055445	Hou et al. (2023)
*Stephanonectriakeithii*	CBS 943.72	OQ429872	OQ430121	Hou et al. (2023)
*Stephanonectriakeithii*	CBS 100007	OQ429871	OQ430120	Hou et al. (2023)
*Mycocitrusodorus*	CBS 100104T	OQ429717	OQ055618	Hou et al. (2023)
*Mycocitrusodorus*	CBS 120610	OQ429715	OQ055616	Hou et al. (2023)
*Mycocitruszonatus*	CBS 400.70	OQ429719	OQ055620	Hou et al. (2023)
*Mycocitrusphyllostachydis*	CBS 330.69	OQ429718	OQ055619	Hou et al. (2023)
*Emericellopsisfuci*	CBS 116467	OQ429564	OQ055477	Hou et al. (2023)
*Emericellopsisfuci*	CBS 485.92	OQ429565	OQ055478	Hou et al. (2023)
*Emericellopsismaritima*	CBS 491.71T	OQ429566	OQ055480	Hou et al. (2023)
*Emericellopsispallida*	CBS 490.71T	OQ429574	OQ055487	Hou et al. (2023)
*Emericellopsisbrunneiguttula*	CBS 111360T	OQ429545	OQ055457	Hou et al. (2023)
*Stanjemoniumgrisellum*	CBS 655.79T	OQ429868	OQ430117	Hou et al. (2023)
*Stanjemoniumochroroseum*	CBS 656.79T	OQ429869	OQ430118	Hou et al. (2023)
*Proliferophialisapiculata*	CBS 303.64T	OQ429796	OQ055692	Hou et al. (2023)
*Proliferophialisapiculata*	CBS 365.64	OQ429797	OQ055693	Hou et al. (2023)
*Acremoniumsubulatum*	CBS 588.73AT	OQ429491	OQ055402	Hou et al. (2023)
*Acremoniumsubulatum*	CBS 115996	OQ429490	OQ055401	Hou et al. (2023)
*Acremoniumaerium*	CBS 189.70T	OQ429441	OQ055352	Hou et al. (2023)
*Acremoniumlongiphialidicum*	CBS 451.70T	OQ429475	OQ055386	Hou et al. (2023)
*Acremoniumpurpurascens*	CBS 149.62T	OQ429485	OQ055396	Hou et al. (2023)
*Acremoniumellipsoideum*	CBS 147433T	OQ429468	OQ055379	Hou et al. (2023)
*Acremoniumellipsoideum*	CBS 147434	OQ429467	OQ055378	Hou et al. (2023)
*Acremoniumbrunneisporum*	CBS 413.76T	OQ429444	OQ055355	Hou et al. (2023)
*Acremoniumbrunneisporum*	CBS 142823	OQ429445	OQ055356	Hou et al. (2023)
*Acremoniummultiramosum*	CBS 147436T	OQ429476	OQ055387	Hou et al. (2023)
*Waltergamsiapilosa*	CBS 124.70T	OQ429949	OQ430199	Hou et al. (2023)
*Waltergamsiapilosa*	CBS 511.82	OQ429948	OQ430198	Hou et al. (2023)
*Waltergamsiaalkalina*	CBS 741.94T	OQ429935	OQ430185	Hou et al. (2023)
*Waltergamsiadimorphospora*	CBS 139050T	LN810515	LN810506	Hou et al. (2023)
*Geosmithiamicrocorthyli*	CCF 3861T	NR_137566	NG_067560	Hou et al. (2023)
*Geosmithiapallidum*	CBS 260.33T	OQ429599	OQ055509	Hou et al. (2023)
*Bulbitheciumspinosum*	CBS 136.33T	OQ429512	OQ055423	Hou et al. (2023)
*Bulbitheciumspinosum*	CBS 915.85	OQ429510	OQ055421	Hou et al. (2023)
*Bulbitheciumarxii*	CBS 737.84T	OQ429505	OQ055416	Hou et al. (2023)
*Bulbitheciumellipsoideum*	CBS 993.69T	OQ429507	OQ055418	Hou et al. (2023)
*Ovicilliumoosporum*	CBS 110151T	OQ429758	OQ055657	Hou et al. (2023)
*Ovicilliumasperulatum*	CBS 130362T	OQ429756	OQ055655	Hou et al. (2023)
*Ovicilliumasperulatum*	CBS 426.95	KU382192	KU382233	Hou et al. (2023)
*Proxiovicilliumblochii*	CBS 427.93T	OQ429816	OQ430079	Hou et al. (2023)
*Proxiovicilliumblochii*	CBS 324.33	OQ429815	OQ430078	Hou et al. (2023)
*Proxiovicilliumlepidopterorum*	CBS 101239T	OQ429817	OQ430080	Hou et al. (2023)
*Hapsidosporaflava*	CBS 596.70T	OQ429649	OQ055555	Hou et al. (2023)
*Hapsidosporaflava*	CBS 316.72	OQ429648	OQ055554	Hou et al. (2023)
*Hapsidosporavariabilis*	CBS 100549T	OQ429663	OQ055569	Hou et al. (2023)
*Hapsidosporastercoraria*	CBS 516.70T	OQ429662	OQ055568	Hou et al. (2023)
*Alloacremoniumhumicola*	CBS 613.82T	OQ429496	OQ055407	Hou et al. (2023)
*Alloacremoniumferrugineum*	CBS 102877T	OQ429495	OQ055406	Hou et al. (2023)
*Stilbocreawalteri*	CBS 144627T	OR050519	OQ430124	Hou et al. (2023)
*Stilbocreamacrostoma*	CBS 114375	OQ429873	OQ430122	Hou et al. (2023)
*Flammocladielladecora*	CBS 142776	MF611693	MF614949	Hou et al. (2023)
*Flammocladiellaaceris*	CBS 138906T	OQ429591	KR611901	Hou et al. (2023)
***Biconidiumsinense***	**GZUIFR 24.013T**	**PQ595985**	**PQ595988**	**This study**
***Biconidiumsinense***	**GZUIFR 24.014**	**PQ595986**	**PQ595989**	**This study**
***Biconidiumsinense***	**GZUIFR 24.015**	**PQ595987**	**PQ595990**	**This study**

Note: T = Ex-type; New isolates in this study are in bold; The line “–” represents the absence of GenBank record. ITS: the internal transcribed spacer region and intervening 5.8S nrRNA; LSU: 28S large subunit.

**Table 2.** Strains of *Didymocyrtis* and corresponding GenBank numbers included in phylogenetic analyses.

Species	Strains	ITS	*tub2*	Reference
*Didymocyrtisbanksiae*	CSN1049	MT813909	–	Monteiro et al. (2022)
*Didymocyrtisbanksiae*	CSN1065	MT813919	–	Monteiro et al. (2022)
*Didymocyrtisbrachylaenae*	CPC 32651	MH327821	MH327896	Monteiro et al. (2022)
*Didymocyrtiscladoniicola*	CBS 131731	KP170644	KP170694	Monteiro et al. (2022)
*Didymocyrtiscladoniicola*	CBS 131732	KP170645	KP170695	Monteiro et al. (2022)
*Didymocyrtisconsimilis*	CBS 129140	MH865190	–	Monteiro et al. (2022)
*Didymocyrtisconsimilis*	CBS 129338	MH865230	–	Monteiro et al. (2022)
*Didymocyrtisepiphyscia*	Freebury 1411	KT383824.1	–	Monteiro et al. (2022)
*Didymocyrtisfoliaceiphila*	CBS 131729	KP170649	KP170699	Monteiro et al. (2022)
*Didymocyrtisfoliaceiphila*	CBS 131730	KP170650	KP170700	Monteiro et al. (2022)
*Didymocyrtismelanelixiae*	Harris 57476 (NY)	KT383831	–	Monteiro et al. (2022)
*Didymocyrtismelanelixiae*	Harris 57475 (NY)	KT383828	–	Monteiro et al. (2022)
*Didymocyrtispini*	CAA 1002 T	MW732246	MW759031	Monteiro et al. (2022)
*Didymocyrtispini*	CAA 1003	MW732247	MW759030	Monteiro et al. (2022)
*Didymocyrtispseudeverniae*	Diederich 17327b	KT383833	–	Monteiro et al. (2022)
*Didymocyrtispseudeverniae*	Diederich 17327a	KT383832	–	Monteiro et al. (2022)
*Didymocyrtisramalinae*	Paul 10i13	KT383839	–	Monteiro et al. (2022)
*Didymocyrtisramalinae*	Paul 27i13	KT383836	–	Monteiro et al. (2022)
*Didymocyrtisseptata*	KNU-JJ-1827	LC552949	–	Monteiro et al. (2022)
*Didymocyrtisslaptonensis*	MoraA (BR)	KT383841	–	Monteiro et al. (2022)
*Didymocyrtistrassii*	AB298	MG519614	–	Monteiro et al. (2022)
*Didymocyrtistrassii*	AB297	MG519613	–	Monteiro et al. (2022)
*Didymocyrtisxanthomendozae*	CBS 129666	KP170651	KP170701	Monteiro et al. (2022)
*Parathyridariaphiladelphi*	CBS 143432	MH107905	–	Monteiro et al. (2022)
***Didymocyrtisshanxiensis***	**GZUIFR 24.004T**	**PQ065635**	**PQ119783**	**This study**
***Didymocyrtisshanxiensis***	**GZUIFR 24.005**	**PQ065636**	**PQ119784**	**This study**

Note: T = Ex-type; New isolates in this study are in bold; The line “–” represents the absence of GenBank record. ITS: the internal transcribed spacer region and intervening 5.8S nrRNA; tub2: β-tubulin.

### ﻿Phylogenetic analysis

**Figure 1.**
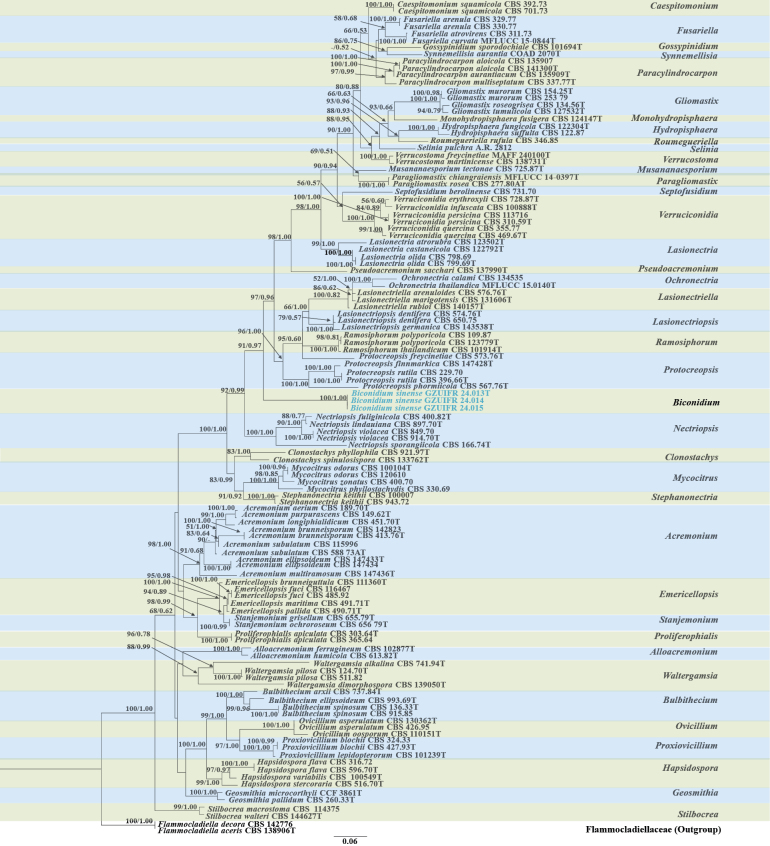
Phylogenetic tree of Bionectriaceae constructed from the dataset of ITS and LSU. Notes: Statistical support values (ML/BI) were shown at nodes. ML bootstrap values ≥50% and posterior probabilities ≥ 0.50 are shown above the internal branches. ‘–’ indicates the absence of statistical support (< 50% for bootstrap proportions from ML analysis; < 0.50 for posterior probabilities from Bayesian analysis). Three new strains are shown in blue font.

The relevant strains sequences were downloaded from GenBank in this paper (Table 1 and Table 2). *Flammocladielladecora* (Wallr.) Lechat & J. Fourn. and *Flammocladiellaaceris* Crous, L. Lombard & R.K. Schumach. were used as the outgroup in phylogenetic tree 1 (Fig. 1). *Parathyridariaphiladelphi* Crous & R.K. Schumach. was used as the outgroup in phylogenetic tree 2 (Fig. 3). The multiple datasets of ITS, LSU and *tub2* were aligned and trimmed in MEGA v.6.06 (Tamura et al. 2013). Using the “Concatenate Sequence” function, the concatenation of loci was conducted in PhyloSuite v.1.16 (Zhang et al. 2020). Then, the phylogenetic construction of each loci dataset was processed by both Maximum Likelihood (ML) and the Bayesian Inference (BI) methods. In ModelFinder, the Akaike Information Criterion correction (AICc) was used for the best-fit substitution model (Kalyaanamoorthy et al. 2017). With 1000 bootstrap tests using the ultrafast algorithm (Minh et al. 2013), the ML analysis was conducted in IQ-TREE v.1.6.11 (Nguyen et al. 2015). The BI analysis was performed in MrBayes v.3.2 (Ronquist et al. 2012) and Markov chain Monte Carlo (MCMC) simulations were used for 2×10^6^ generations. Using FigTree version 1.4.3, the phylogenetic trees were visualized and edited in Microsoft PowerPoint.

## ﻿Results

### ﻿Phylogenetic analysis

In this study, using ITS sequences, our five isolates were identified and assigned to potential genera and species based on a BLASTn in NCBI. Five strains belonging to Bionectriaceae or *Didymocyrtis* were screened and tested for further identification through morphological characterization and phylogenetic analyses. Using ML and BI analyses, the two phylogenetic trees were consistent and supported strongly in branches. The ML analysis for the combined dataset provided the best scoring tree. The concatenated sequences of Fig. 1 and Fig. 3 included 90 and 16 taxa, respectively. The dataset in Fig. 1 was composed of ITS (1–382 bp) and LSU (383–782 bp) sequence data. The dataset in Fig. 3 was composed of ITS (1–402 bp) and *tub2* (403–731 bp) sequence data.

The phylogeny shows that each genus clusters into a monophyletic clade, and three strains of the genus *Biconidium* clustered in a well-separated clade, with a high support value (ML/BI 100/1) (Fig. 1). Two strains of the genus *Didymocyrtis* also clustered together, with a high support value (ML/BI 98/1) (Fig. 3). Therefore, a new genus, *Biconidium* H.Y. Wang & Y.F. Han, is introduced, and *Biconidiumsinense* H.Y. Wang & Y.F. Han and *Didymocyrtisshanxiensis* H.Y. Wang & Y.F. Han as new species are proposed according to the phylogenetic analysis.

#### ﻿Sordariomycetes O.E. Erikss. & Winka

**Hypocreales Lindau**

**Bionectriaceae Samuels & Rossman**

##### ﻿ Biconidium

Taxon classificationFungiHypocrealesBionectriaceae

H.Y. Wang & Y.F. Han gen. nov.

01EEEA05-F306-5CD6-92CD-2E62F6A4778B

MB857281

###### Etymology.

Referring to the bicellular conidia.

###### Description.

***Mycelium*** hyaline, septate, smooth, thin-walled. ***Conidiophores*** hyaline, septate, smooth-walled, solitary, straight, (sub-)erect, arising directly from hyphae, unbranched or branched, bearing 1–5 levels with 1–6 phialides per node. ***Conidiogenous cells*** enteroblastic, monophialidic, lateral or terminal, awl-shaped, hyaline, smooth, with globose to cylindriform thickening at conidiogenous loci. ***Conidia*** bicellular, podiform, unsymmetrically at both ends, hyaline, thick-walled, smooth, arranged in slimy heads. Chlamydospores and sexual morph absent.

###### Type species.

*Biconidiumsinense* H.Y. Wang & Y.F. Han

###### Notes.

Three isolates from green soil of sewage treatment plant clearly form an independent clade on the ITS and LSU tree (Fig. 1), and are phylogenetically segregated from other genera, representing the new species with conidiogenous cells with globose to cylindriform thickening at conidiogenous loci and podiform conidia arranged in slimy heads. Therefore, we introduce *Biconidium* as a new genus to accommodate this species.

##### ﻿ Biconidium sinense

Taxon classificationFungiHypocrealesBionectriaceae

H.Y. Wang & Y.F. Han sp. nov.

211757A1-26E1-5349-9F05-A5C09B8DB8CE

MB857282

Fig. 2

###### Etymology.

Referring to China where the species was isolated.

###### Type.

China • Zhejiang Province, Hangzhou City, sewage treatment plant (30°10'53"N, 120°10'2"E), soil, August 2021, Yulian Ren, ex-type culture GZUIFR 24.013, dried holotype GZAC 24.013. ITS sequences, GenBank PQ595985; LSU sequences, GenBank PQ595988.

###### Description.

Culture characteristics (7 days of incubation at 25 °C): Colony on PDA, 20–30 mm diam., fleshy, plicated, beige (RAL1001) at the center, villiform, traffic white (RAL 9016) at the edge, reverse, light lvory (RAL1015) at the center, cream (RAL9001) at the edge, nearly round, margin partial; Colony on MEA, 25–30 mm diam., flocculence, traffic white (RAL 9016), reverse, broom yellow (RAL1032), margin entire, round. Colony on OA, 30–35 mm diam., thin, short villous, signal white (RAL9003), reverse, cream (RAL9001), margin entire, round.

**Figure 2.**
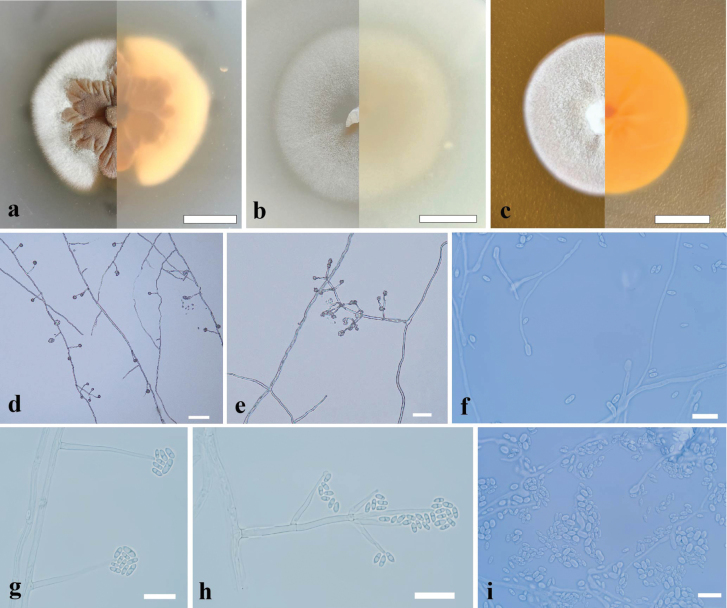
Morphological characteristics of *Biconidiumsinense* sp. nov. **a–c** front and reverse of colony on PDA, OA and MEA after 7 days at 25 °C **d, e** conidiophores and conidial heads **f–h** conidiophores and conidia **i** conidia. Scale bars: 10 mm (**a–c**); 50 μm (**d**); 20 μm (**e**); 10 μm (**f–i**).

On PDA, ***Mycelium*** hyaline, septate, smooth, thin-walled 1.2–2.7 μm wide. ***Conidiophores*** hyaline, septate, smooth, solitary, straight, (sub-)erect, arising directly from hyphae, branched or unbranched, bearing 1–5 levels with 1–6 phialides, 1–3 septate at base or middle, 20–52 μm long, 1.5–2.7 μm wide at base. ***Phialides*** lateral or terminal, from the conidiophores or directly from the mycelia, awl-shaped, hyaline, smooth-walled, 9.5–35 μm long, 1–2.3 μm wide at base, with globose to cylindriform thickening at conidiogenous loci. polyphialides not observed. ***Conidia*** podiform, 1-septate, 2.5–6.0 × 1.0–3.0 μm (mean ± SD = 3.5 ± 1.0 × 2.0 ± 0.5 μm, n = 30), center-empty, unsymmetrically at both ends, apex angular, base subobtuse, hyaline, thick-, smooth-walled, arranged in slimy heads. Chlamydospores and sexual morph not observed.

**Figure 3.**
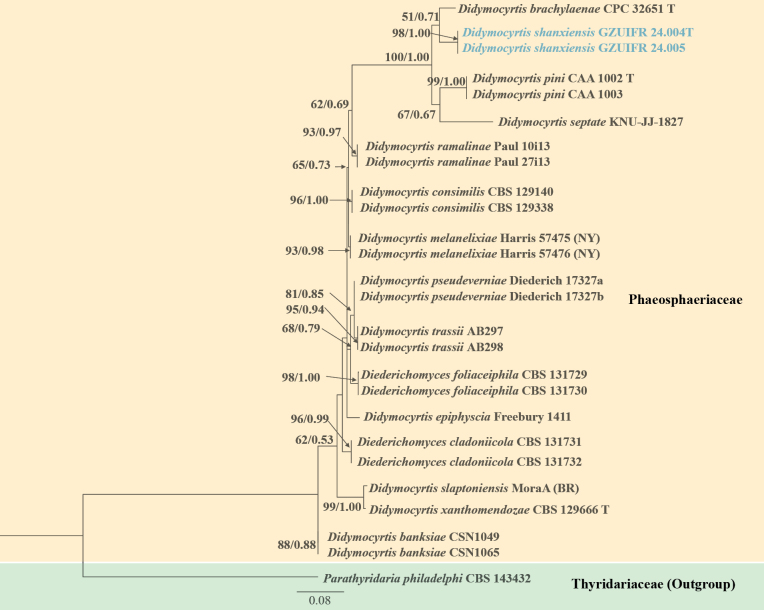
Phylogenetic tree of the genus *Didymocyrtis* constructed from the dataset of ITS and *tub2*. Notes: Statistical support values (ML/BI) were shown at nodes. ML bootstrap values ≥50% and posterior probabilities ≥ 0.50 are shown above the internal branches. Two new strains are shown in blue font.

###### Additional specimens examined.

China • Zhejiang Province, Hangzhou City, sewage treatment plant (30°10'53"N, 120°10'2"E), soil, August 2021, living cultures GZUIFR 24.014 (ITS sequences, GenBank PQ595986; LSU sequences, GenBank PQ595989), GZUIFR 24.015 (ITS sequences, GenBank PQ595987; LSU sequences, GenBank PQ595990).

###### Notes.

Phylogenetically, our three strains (GZUIFR 24.013, GZUIFR 24.014 and GZUIFR 24.015) can apparently separate with other species in Bionectriaceae, and clustered in a single clade with a high support value (BI pp = posterior probability 1, ML BS 100) (Fig. 1). *Biconidiumsinense* is distinguished from other species of Bionectriaceae by conidiogenous cells with globose to cylindriform thickening at conidiogenous loci, and podiform conidia arranged in slimy heads in the morphological characteristics.

#### ﻿Dothideomycetes O.E. Erikss. & Winka

**Pleosporales Luttr. ex M.E. Barr**

**Phaeosphaeriaceae M.E. Barr**

***Didymocyrtis* Vain.**

##### ﻿ Didymocyrtis shanxiensis

Taxon classificationFungiPleosporalesPhaeosphaeriaceae

H.Y. Wang & Y.F. Han sp. nov.

796B50E8-9E92-5008-94EC-F3FCB636F9FC

MB857280

Fig. 4

###### Etymology.

shanxiensis, referring to Shanxi province where the type locality was isolated.

###### Type.

China • Shanxi Province, Datong City, sewage treatment plant (40°2'42"N, 113°20'48"E), soil, August 2021, Yulian Ren, ex-type culture GZUIFR 24.004, dried holotype GZAC 24.004. ITS sequences, GenBank PQ065635; *tub2* sequences, GenBank PQ119783.

###### Description.

Culture characteristics (7 days of incubation at 25 °C): Colony on PDA, 30–35 mm diam., thin, villiform, cream (RAL9001), reverse cream (RAL9001), regular in the margin; Colony on MEA, 20–25 mm diam., thick, villiform, light lvory (RAL1015), reverse dahlia yellow (RAL1033), regular in the margin; Colony on OA, 30–35 mm diam., texture velvety, olive yellow (RAL1020), reverse stone gray (RAL7030), regular in the margin. Black spots produced after incubating 15 days on PDA.

**Figure 4.**
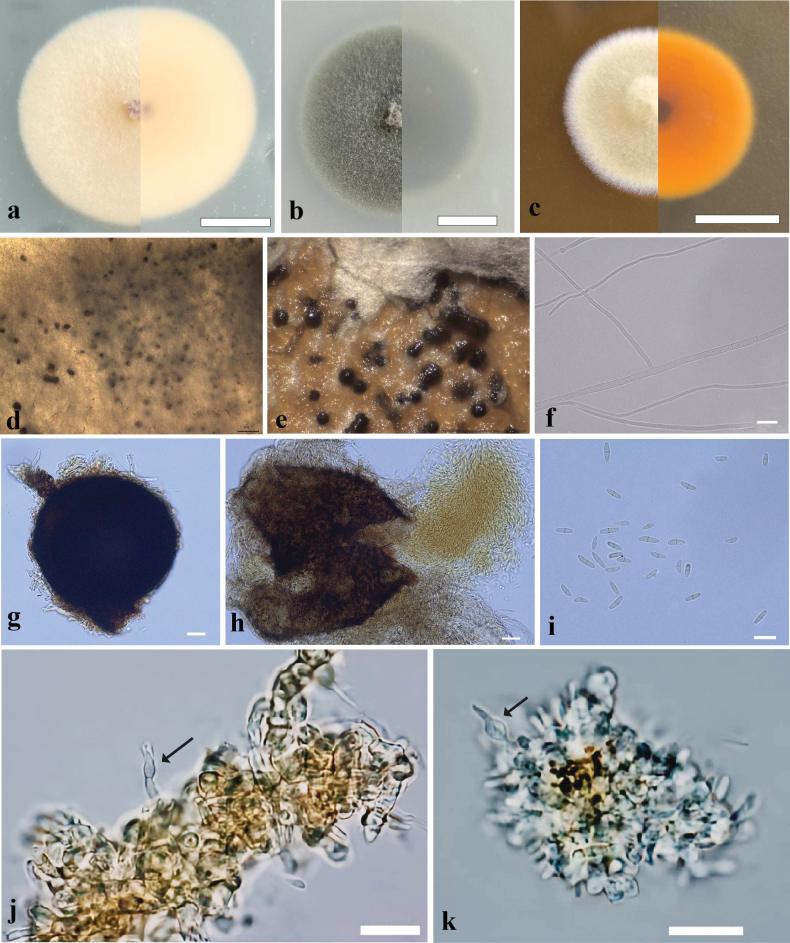
Morphological characteristics of *Didymocyrtisshanxiensis* sp. nov. **a–c** front and reverse of colony on PDA, OA and MEA after 7 days at 25 °C **d, e** conidiomata on culture **f** hyphae **g, h** conidiomata and ruptured conidiomata with conidia mass **i** conidia **j, k** conidiogenous cells. Scale bars: 10 mm (**a–c**); 20 μm (**g, h**); 10 μm (**i**); 20 μm (**j, k**).

On PDA medium after 30 days of incubation at 25 °C, ***Hyphae*** septate, hyaline, smooth, thick-walled, 1.0–2.5 μm wide. ***Conidiomata*** submersed, brown to black, globose, 150–250 µm diam. ***Conidiophores*** reduced to conidiogenous cells. ***Conidiogenous cells*** globose to subglobose, also ampulliform, aseptate, hyaline, smooth, thick-walled, 4.5–10.0 × 2.0–6.0 μm (mean ± SD = 7.0 ± 1.9 × 3.5 ± 1.0 μm, n = 15). ***Conidia*** abundant, cymbiform mostly, brown, smooth, apex subobtuse, base truncate, 1-septate, 5.0–11.0 × 1.5–3.0 μm (mean ± SD = 7.5 ± 1.6 × 2.0 ± 0.4 μm, n = 30).

###### Additional specimens examined.

China • Shanxi Province, Datong City, sewage treatment plant (40°2'42"N, 113°20'48"E), soil, August 2021, living cultures GZUIFR 24.005. ITS sequences, GenBank PQ065636; *tub2* sequences, GenBank PQ119784.

###### Notes.

Twenty-nine species of the genus *Didymocyrtis* are recorded in the Index Fungorum. However, the DNA sequence data of fifteen species have no records in NCBI database. Phylogenetically, our two strains (GZUIFR 24.004 and GZUIFR 24.005) clustered in a single clade with a high support value (ML/BI 98/1) (Fig. 3). In the phylogenetic tree, although our new species *D.shanxiensis* and *Didymocyrtisbrachylaenae* Crous are closely related species, they were obviously different in morphological characteristics. *Didymocyrtisshanxiensis*, having conidiophores reduced to conidiogenous cells, globose to subglobose and ampulliform conidiogenous cells, and cymbiform conidia, can be distinguished from *D.brachylaenae* with subcylindrical and branched conidiophores, lining the inner cavity and ampulliform to doliiform conidiogenous cells, and fusoidellipsoid to subcylindrical conidia (Crous et al. 2018).

## ﻿Discussion

Hou et al. (2023) revaluated acremonium-like fungi in Hypocreales, and found most species of *Acremoniums. lat.* grouped in genera of Bionectriaceae. Therefore, the phylogenetic tree of Bionectriaceae is provided based on multi-locus (ITS, LSU, *rpb2*, *tef-1α*) DNA sequencing analyses to accommodate 183 species and 39 genera including 10 new genera. In this study, employing ITS and LSU sequences can well distinguish the species of Bionectriaceae. From the phylogenetic tree (Fig. 1), three strains of our new species *Biconidiumsinense* cluster in a well-separated clade with a high support value (ML/BI 100/1). Meanwhile, *B.sinense* having conidiogenous cells with globose to cylindriform thickening at conidiogenous loci, and podiform conidia arranged in slimy heads differs from all other species of Bionectriaceae. Therefore, *Biconidium* is introduced to accommodate a new species *B.sinense* combined with phylogenetic and morphological analyses. Bionectriaceae are including both sexual morphs and asexual taxa (Hou et al. 2023). Species of the Bionectriaceae are mostly found in terrestrial or freshwater environments, with fewer commonly found in marine habitats, and they are common coprophilous, corticolous, fungicolous, lichenicolous or herbicolous (Zhao et al. 2023). In this study, our three strains of *B.sinense* were isolated from green soils of sewage treatment plant.

In this study, although *D.shanxiensis*, *D.brachylaenae*, *D.pini* and *D.septata* clustered as the sister subclades, they were obviously different in morphological characteristics. Morphologically, the main characteristics of *D.shanxiensis* are having globose conidiomata, conidiophores reduced to conidiogenous cells, globose to subglobose and ampulliform conidiogenous cells, and the smaller size of cymbiform conidia (mean size = 7.5 × 2.0 μm). While, *D.brachylaenae* can be distinguished from *D.shanxiensis* by having subcylindrical and branched conidiophores, and fusoidellipsoid to subcylindrical conidia (Crous et al. 2018); *Didymocyrtispini* can be distinguished from *D.shanxiensis* by having fusiform conidia (mean size = 8.5 × 2.4 μm) (Monteiro et al. 2022); *Didymocyrtisseptate* differed from *D.shanxiensis* by having irregular conidiomata, and fusiform, clavate to subcylindrical conidia (mean size = 8.2 × 2.3 μm) (Das et al. 2021). At the same time, *D.shanxiensis* has a clear morphological difference from fifteen species without DNA sequence data (Joshi et al. 2024), so it is proposed as a new species in the genus *Didymocyrtis*. Up to now, the most species of *Didymocyrtis* are lichenicolous fungi living parasitic life-styleare (Ertz et al. 2015; Suija et al. 2021). Some *Didymocyrtis* spp. are pathogenic fungi and saprophytic fungi. For example, *D.brachylaenae* and *D.pini* as pathogeny live on plant leaves (Crous et al. 2018; Monteiro et al. 2022), and *D.septata* is saprophytic in containing plant soil (Das et al. 2021). Our two strains of new species were also isolated from green land soil in this study and possible to be saprophytic. Presently, this genus includes twenty-nine species in the Index Fungorum (http://www.indexfungorum.org/Names/Names.asp, retrieval on 10 January 2025). Here, together with *D.shanxiensis*, the genus *Didymocyrtis* has a total of thirty species.

Though two new species were reported in this study, we believed that more new taxa will be found and reported from the various soil habitats, which are deserving to be explored in the future.

## Supplementary Material

XML Treatment for Biconidium

XML Treatment for Biconidium sinense

XML Treatment for Didymocyrtis shanxiensis

## ﻿Additional information

### Author ORCIDs

Hai-Yan Wang https://orcid.org/0000-0001-9190-0490

Chunbo Dong https://orcid.org/0000-0001-7074-5700

Yan-Wei Zhang https://orcid.org/0000-0003-1251-5821

Wan-Hao Chen https://orcid.org/0000-0001-7240-6841

Yan-Feng Han https://orcid.org/0000-0002-8646-3975

### Data availability

All of the data that support the findings of this study are available in the main text.

## References

[B1] Akilandeswari P Pradeep B (2016) Exploration of industrially important pigments from soil fungi. Applied Microbiology and Biotechnology 100(4): 1631–1643. 10.1007/s00253-015-7231-8 26701360

[B2] Barr ME (1979) A classification of Loculoascomycetes. Mycologia 71: 935–957. 10.1080/00275514.1979.12021099

[B3] Buczkowski G Richmond DS (2012) The effect of urbanization on ant abundance and diversity: A temporal examination of factors affecting biodiversity. PLOS ONE 7(8): e41729. 10.1371/journal.pone.0041729 PMC3410901 22876291

[B4] Crous PW Wingfield MJ Burgess TI Hardy GSJ Gené J Guarro J Baseia IG García D Gusmão LFP Souza-Motta CM Thangavel R Adamčík S Barili A Barnes CW Bezerra JDP Bordallo JJ Cano-Lira JF de Oliveira RJV Ercole E Hubka V Iturrieta-González I Kubátová A Martín MP Moreau PA Morte A Ordoñez ME Rodríguez A Stchigel AM Vizzini A Abdollahzadeh J Abreu VP Adamčíková K Albuquerque GMR Alexandrova AV Álvarez Duarte E Armstrong-Cho C Banniza S Barbosa RN Bellanger JM Bezerra JL Cabral TS Caboň M Caicedo E Cantillo T Carnegie AJ Carmo LT Castañeda-Ruiz RF Clement CR Čmoková A Conceição LB Cruz RHSF Damm U da Silva BDB da Silva GA da Silva RMF de A Santiago ALCM de Oliveira LF de Souza CAF Déniel F Dima B Dong G Edwards J Félix CR Fournier J Gibertoni TB Hosaka K Iturriaga T Jadan M Jany JL Jurjević Ž Kolařík M Kušan I Landell MF Leite Cordeiro TR Lima DX Loizides M Luo S Machado AR Madrid H Magalhães OMC Marinho P Matočec N Mešić A Miller AN Morozova OV Neves RP Nonaka K Nováková A Oberlies NH Oliveira-Filho JRC Oliveira TGL Papp V Pereira OL Perrone G Peterson SW Pham THG Raja HA Raudabaugh DB Řehulka J Rodríguez-Andrade E Saba M Schauflerová A Shivas RG Simonini G Siqueira JPZ Sousa JO Stajsic V Svetasheva T Tan YP Tkalčec Z Ullah S Valente P Valenzuela-Lopez N Abrinbana M Viana Marques DA Wong PTW Xavier de Lima V Groenewald JZ (2018) Fungal planet description sheets: 716–784. Persoonia. Molecular Phylogeny and Evolution of Fungi 40: 240–393. 10.3767/persoonia.2018.40.10 PMC6146637 30505003

[B5] Das K Lee SY Jung HY (2021) Morphology and phylogeny of two novel species within the Class Dothideomycetes collected from Soil in Korea. Mycobiology 49: 15–23. 10.1080/12298093.2020.1838114 PMC7832580 33536809

[B6] Desa UN (2019) World population prospects 2019: Highlights. New York (US). United Nations Department for Economic and Social Affairs 11(1): 125.

[B7] Ertz D Diederich P Lawrey JD Berger F Freebury CE Coppins B Gardiennet A Hafellner J (2015) Phylogenetic insights resolve Dacampiaceae (Pleosporales) as polyphyletic: *Didymocyrtis* (Pleosporales, Phaeosphaeriaceae) with *Phoma*-like anamorphs resurrected and segregated from *Polycoccum* (Trypetheliales, Polycoccaceae fam. nov.). Fungal Diversity 74: 53–89. 10.1007/s13225-015-0345-6

[B8] Grimm NB Faeth SH Golubiewski NE Redman CL Wu J Bai X Briggs JM (2008) Global change and the ecology of cities. Science 319: 756–760. 10.1126/science.1150195 18258902

[B9] Guo H (2024) Effects of different stubble and nitrogen application rates on yield traits and soil microorganisms of winter wheat. Master Thesis, Henan Agricultural University, Zhengzhou, Chain.

[B10] Guo CJ Zhang LR Shen RQ Xu BL (2017) Diversity of rhizosphere soil fungi in sand‐fixation plants in Tengger Desert of Ningxia Autonomous Region. Junwu Xuebao 36(5): 552–562. 10.13346/j.mycosystema.160083

[B11] Hou Y Zhou HP Zhang C (2014) Effects of urbanization on community structure of soil microorganism. Shengtai Huanjing Xuebao 23(7): 1108–1112. 10.16258/j.cnki.1674-5906.2014.07.002

[B12] Hou LW Giraldo A Groenewald JZ Rämä T Summerbell RC Zang P Cai L Crous PW (2023) Redisposition of *acremonium*-like fungi in Hypocreales. Studies in Mycology 105: 23–203. 10.3114/sim.2023.105.02 38895703 PMC11182610

[B13] Joshi Y Bisht S Bansal P (2024) *Didymocyrtispertusariae*: A new species from Central Himalaya, India and a worldwide key to all recognized *Didymocyrtis* (Phaeosphaeriaceae; Pleosporales) species. Journal of Asia-Pacific Biodiversity 17(3): 550–561. 10.1016/j.japb.2024.04.005

[B14] Kalyaanamoorthy S Minh BQ Wong TKF von Haeseler A Jermiin LS (2017) ModelFinder: Fast model selection for accurate phylogenetic estimates. Nature Methods 14(6): 587–589. 10.1038/nmeth.4285 28481363 PMC5453245

[B15] Lawrey JD Diederich P Nelsen MP Freebury C Van den Broeck D Sikaroodi M Ertz D (2012) Phylogenetic placement of lichenicolous *Phoma* species in the Phaeosphaeriaceae (Pleosporales, Dothideomycetes). Fungal Diversity 55: 195–213. 10.1007/s13225-012-0166-9

[B16] Li X Zhang ZY Chen WH Liang JD Huang JZ Han YF Liang ZQ (2022a) A new species of *Arthrographis* (Eremomycetaceae, Dothideomycetes), from the soil in Guizhou, China. Phytotaxa 538(3): 175–181. 10.11646/phytotaxa.538.3.1

[B17] Li X Zhang ZY Ren YL Chen WH Liang JD Pan JM Huang JZ Liang ZQ Han YF (2022b) Morphological characteristics and phylogenetic evidence reveal two new species of *Acremonium* (Hypocreales, Sordariomycetes). MycoKeys 91: 85–96. 10.3897/mycokeys.91.86257 36760887 PMC9849060

[B18] Löbl I Klausnitzer B Hartmann M Krell FT (2023) The silent extinction of species and taxonomists—An appeal to science policymakers and legislators. Diversity 15(10): 1053. 10.3390/d15101053

[B19] Lu M (2018) Effects of wetlands degradation on structure and biodiversity of soil microbial community in Napahai plateau wetlands. PhD Thesis, Beijing Forestry University, Beijing, China.

[B20] Ma CX (2023) The Influence of Moso Banboo Forest and Chinese Fir Forest on Soil Microorganisms of Typical Subtropical. Shandong Nongye Daxue Xuebao 54(3): 391–396. 10.3969/j.issn.1000-2324.2023.03.009 [Natural Science Edition]

[B21] Ma YS Lu ZY Yang HL Zhang ZB Yan RM Jiang YM Zhu D (2021) Comparison of soil microbial community structure in different seasons in Longgong Cave, Jiangxi Province. Journal of Jiangxi Normal University 45(2): 162–171. 10.16357/j.cnki.issn1000-5862.2021.02.09 [Natural Science]

[B22] Minh BQ Nguyen MAT von Haeseler A (2013) Ultrafast approximation for phylogenetic bootstrap. Molecular Biology and Evolution 30(5): 1188–1195. 10.1093/molbev/mst024 23418397 PMC3670741

[B23] Monteiro P Gonçalves MF Pinto G Silva B Martín-García J Diez JJ Alves A (2022) Three novel species of fungi associated with pine species showing needle blight-like disease symptoms. European Journal of Plant Pathology 162: 183–202. 10.1007/s10658-021-02395-5

[B24] Nguyen LT Schmidt HA von Haeseler A Minh BQ (2015) IQ-TREE: A fast and effective stochastic algorithm for estimating maximum-likelihood phylogenies. Molecular Biology and Evolution 32(1): 268–274. 10.1093/molbev/msu300 25371430 PMC4271533

[B25] Nugent A Allison SD (2022) A framework for soil microbial ecology in urban ecosystems. Ecosphere 13(3): e3968. 10.1002/ecs2.3968

[B26] O’Donnell K Cigelnik E (1997) Two divergent intragenomic rDNA ITS2 types within a monophyletic lineage of the fungus *Fusarium* are nonorthologous. Molecular Phylogenetics and Evolution 7(1): 103–116. 10.1006/mpev.1996.0376 9007025

[B27] Rai PK Rai A Singh S (2018) Change in soil microbial biomass along a rural-urban gradient in Varanasi (U.P., India). Geology, Ecology, and Landscapes 2: 15–21. 10.1080/24749508.2018.1438743

[B28] Ren YL Zhang ZY Chen WH Liang JD Han YF Liang ZQ (2022) Morphological and phylogenetic characteristics of *Phaeomycocentrosporaxinjangensis* (Pleosporales, Dothideomycetes), a new species from China. Phytotaxa 558(1): 125–132. 10.11646/phytotaxa.558.1.9

[B29] Ronquist F Teslenko M van der Mark P Ayres DL Darling A Höhna S Larget B Liu L Suchard MA Huelsenbeck JP (2012) MrBayes 3.2: Efficient bayesian phylogenetic inference and model choice across a large model space. Systematic Biology 61(3): 539–542. 10.1093/sysbio/sys029 22357727 PMC3329765

[B30] Rossman AY Samuels GJ Rogerson CT Lowen R (1999) Genera of Bionectriaceae, Hypocreaceae and Nectriaceae (Hypocreales, Ascomycetes). Studies in Mycology 42: 1–248.

[B31] Song JQ Yin YL Zhang W Liu Y Sui QQ Huo JY Zheng WX Li SX (2024) Characteristics of spatial differentiation of soil microbial communities in degraded grassland on the “black soil beaches” of Qinghai Plateau. Shengtai Huanjing Xuebao 33(11): 1696–1707. 10.16258/j.cnki.1674-5906.2024.11.004

[B32] Spegazzini C (1919) Fungi Costaricenses Nonnulli. Fungi Costaricenses Nonnulli. Boletin de la Academia Nacional de Ciencias en Córdoba 23: 563.

[B33] Suija A Delhoume A Poumarat S Diederich P (2021) *Didymocyrtismicroxanthoriae* (Phaeosphaeriaceae, Dothideomycetes), a new lichenicolous fungus from France. Bulletin dela Société des naturalistes luxembourgeois 123: 129–136.

[B34] Tamura K Stecher G Peterson D Filipski A Kumar S (2013) MEGA6: Molecular evolutionary genetics analysis version 6.0. Molecular Biology and Evolution 30(12): 2725–2729. 10.1093/molbev/mst197 24132122 PMC3840312

[B35] Trakunyingcharoen T Lombard L Groenewald JZ Cheewangkoon R Toanun C Alfenas AC Crous PW (2014) Mycoparasitic species of *Sphaerellopsis*, and allied lichenicolous and other genera. IMA Fungus 5(2): 391–414. 10.5598/imafungus.2014.05.02.05 25734030 PMC4329322

[B36] Vainio EA (1921) Lichenographia Fennia I. Acta Societatis pro Fauna et Flora Fennica. 49(2): 1–274.

[B37] Wang HY (2024) Study on the diversity and keratinophilic functional strains of heat-tolerant fungi in zoo soils. Master Thesis, Guizhou University, Guiyang, China.

[B38] Wang MX Liu KY Xing YJ (2018) Association analysis of soil microorganism and plant species diversity under climate change. Zhongguo Nongxue Tongbao 34(20): 111–117.

[B39] Wang XW Han PJ Bai FY Luo A Bensch K Meijer M Kraak B Han DY Sun BD Crous PW Houbraken J (2022) Taxonomy, phylogeny and identification of Chaetomiaceae with emphasis on thermophilic species. Studies in Mycology 101(1): 121–243. 10.3114/sim.2022.101.03 36059895 PMC9365047

[B40] Wang HY Zhang ZY Ren YL Shao QY Li X Chen WH Liang JD Liang ZQ Han YF (2023) *Multiverrucasinensis* gen. nov., sp. nov., a thermotolerant fungus isolated from soil in China. International Journal of Systematic and Evolutionary Microbiology 73(2): 005734. 10.1099/ijsem.0.005734 36815560

[B41] Wang HY Li X Dong CB Zhang YW Chen WH Liang JD Han YF (2024) Two new species of Sordariomycetes (Chaetomiaceae and Nectriaceae) from China. MycoKeys 102: 301–315. 10.3897/mycokeys.102.114480 38495535 PMC10940860

[B42] White TJ Bruns T Lee S Taylor J (1990) Amplification and direct sequencing of fungal ribosomal RNA genes for phylogenetics. PCR protocols: a guide to methods and applications 18(1): 315–322. 10.1016/B978-0-12-372180-8.50042-1

[B43] Woudenberg JHC Aveskamp MM de Gruyter J Spiers AG Crous PW (2009) Multiple *Didymella* teleomorphs are linked to the *Phomaclematidina* morphotype. Persoonia 22(1): 56–62. 10.3767/003158509X427808 20198138 PMC2789541

[B44] Yan B Lu Q Xia S Li JS (2022) An overview of advances in soil microbial diversity of urban environment. Shengwu Duoyangxing 30: 22186. 10.17520/biods.2022186

[B45] Yogabaanu U Weber JFF Convey P Rizman-Idid M Alias SA (2017) Antimicrobial properties and the influence of temperature on secondary metabolite production in cold environment soil fungi. Polar Science 14: 60–67. 10.1016/j.polar.2017.09.005

[B46] Zhang D Gao FL Jakovlić I Zou H Zheng J Li WX Wang GT (2020) PhyloSuite: An integrated and scalable desktop platform for streamlined molecular sequence data management and evolutionary phylogenetics studies. Molecular Ecology Resources 20(1): 348–355. 10.1111/1755-0998.13096 31599058

[B47] Zhang ZY Shao QY Li X Chen WH Liang JD Han YF Huang JZ Liang ZQ (2021) Culturable fungi from urban soils in China I: Description of 10 new taxa. Microbiology Spectrum 9: e00867–e21. 10.1128/Spectrum.00867-21 PMC8510251 34612666

[B48] Zhang ZY Li X Chen WH Liang JD Han YF (2023) Culturable fungi from urban soils in China II, with the description of 18 novel species in Ascomycota (Dothideomycetes, Eurotiomycetes, Leotiomycetes and Sordariomycetes). MycoKeys 98: 167–220. 10.3897/mycokeys.98.102816 37425100 PMC10326621

[B49] Zhang ZY Pan H Tao G Li X Han YF Feng Y Tong SQ Ding CY (2024) Culturable mycobiota from Guizhou wildlife park in China. Mycosphere: Journal of Fungal Biology 15(1): 654–763. 10.5943/mycosphere/15/1/5

[B50] Zhao D Li F Wang RS Yang QR Ni HS (2012) Effect of soil sealing on the microbial biomass, N transformation and related enzyme activities at various depths of soils in urban area of Beijing, China. Journal of Soils and Sediments 12: 519–530. 10.1007/s11368-012-0472-6

[B51] Zhao L Groenewald JZ Hernández-Restrepo M Schroers HJ Crous PW (2023) Revising *Clonostachys* and allied genera in Bionectriaceae. Studies in Mycology 105: 205–266. 10.3114/sim.2023.105.03 38895704 PMC11182609

